# Supplementary material for: Content-rich biological network constructed by mining PubMed abstracts
Source: BMC Bioinformatics. 2004 Oct 8;5:147. doi: 10.1186/1471-2105-5-147 (PMC528731; doi:10.1186/1471-2105-5-147)
Supplement: Additional File 2 — The original results of the above study (non-essential files are deleted to keep the file size under the limit set by BMC bioinformatics). [file 1471-2105-5-147-S2.bz2 › chilibotAdditionalFile2/dip20/707ID9482750E18044/html/MUTL_UVRD.html]

 


 **MUTL** and **UVRD** 
  
Found 29 abstracts in PubMed, retrieved 20.  
 

 What does Google say? 
 PDF only 
| .edu only 

---

**Interactive relationship** (e.g. stimulation, inhibition, etc)

**Neutral relationship**- The results indicate that  **MutL**  binds DNA and loads  **UvrD**  onto the DNA substrate.  Ref: 10984488 J Biol Chem, 2000
- Escherichia coli  **MutL**  loads DNA helicase II  [ **UVRD** ]  onto DNA.  Ref: 10984488 J Biol Chem, 2000
- MutS and  **MutL**  activate DNA helicase II  [ **UVRD** ]  in a mismatch dependent manner.  Ref: 9535910 J Biol Chem, 1998
- Previous studies have shown that  **MutL**  physically interacts with  **UvrD**  DNA helicase II  [ **UVRD** ]  Hall, M.  Ref: 10984488 J Biol Chem, 2000
- Evidence for a physical interaction between the Escherichia coli methyl directed mismatch repair proteins  **MutL**  and  **UvrD** .  Ref: 9482750 EMBO J, 1998
- Screening of an E.coli genomic library revealed a potential interaction between  **UvrD**  and  **MutL** , a component of the methyl directed mismatch repair pathway.  Ref: 9482750 EMBO J, 1998
- On the other hand, both the N and C termini of  **UvrD**  were required for interaction with  **MutL** .  Ref: 9482750 EMBO J, 1998
- Deletion analysis demonstrated that the C terminal 218 amino acids residues 398 615 of  **MutL**  were sufficient to produce the two hybrid interaction with  **UvrD** .  Ref: 9482750 EMBO J, 1998
- A two hybrid screen previously identified a physical interaction between  **MutL**  and  **UvrD** .  Ref: 9880500 J Biol Chem, 1999
- All possible pairwise combinations of  **UvrD** ,  **MutL** , MutS, and MutH were tested using the yeast two hybrid system to identify potential interactions involving mismatch repair proteins.  Ref: 9880500 J Biol Chem, 1999
- As judged by protein affinity chromatography,  **MutL**  and  **MutL**  E32K both support formation of ternary complexes that also contain MutS and MutH or MutS and DNA helicase II  [ **UVRD** ] .  Ref: 10734142 J Biol Chem, 2000
- This phenotype is suppressed by a  **uvrD**  mutU defect, and partially suppressed by mutH,  **mutL** , or mutS mutations.  Ref: 11381137 Proc Natl Acad Sci U S A, 2001
- between  **MutL**  and  **UvrD**  are both important for stimulation of  **UvrD**  catalyzed unwinding.  Ref: 10984488 J Biol Chem, 2000

**Non-interactive relationship** (e.g. studied together, co-existance, homology, etc.)

- Escherichia coli MutS,  **MutL** , and DNA helicase II  [ **UVRD** ]  are sufficient to initiate mismatch dependent unwinding of an incised heteroduplex Yamaguchi, M.  Ref: 9535911 J Biol Chem, 1998
- The increases in spontaneous reversion of histidine mutations were first measured in isogenic strains carrying mismatch repair defective mutH,  **mutL** , mutS, or  **uvrD**  alleles, relative to their mismatch repair proficient counterparts.  Ref: 9685594 Mutat Res, 1998
- Deletion rates were enhanced 30 to 50 fold by the mismatch repair defects, mutS,  **mutL**  and  **uvrD** , and 250 fold by the ssb 3 allele, but the effect of the mismatch defects was canceled by the delta recA allele.  Ref: 9055067 Genetics, 1997
- We screened the following host mutator mutations for effects on spontaneous mutation rates in T4 mutT destruction of aberrant dGTPs, polA, polB and polC DNA polymerases, dnaQ exonucleolytic proofreading, mutH, mutS,  **mutL**  and  **uvrD**  methyl directed DNA mismatch repair, mutM and mutY excision repair of oxygen damaged DNA, mutA function unknown, and topB and osmZ affecting DNA topology.  Ref: 7851754 Genetics, 1994
- Here, we report the cloning and sequencing of two additional P. aeruginosa mismatch repair genes and the characterization, by complementation of deficient strains, of these two putative P. aeruginosa mismatch repair genes  **mutL**  and  **uvrD** .  Ref: 11952911 Mol Microbiol, 2002
- **MutL**  does not clamp  **UvrD**  onto the substrate.  Ref: 10984488 J Biol Chem, 2000
- MutS,  **MutL** , and DNA helicase II  [ **UVRD** ]  are required for the mismatch provoked excision step that occurs during Escherichia coli methyl directed mismatch repair.  Ref: 9535910 J Biol Chem, 1998
- The in vitro activity was also dependent on products of mutH,  **mutL** , mutS, and  **uvrD**  loci and was equally efficient on nucleotide insertions and deletions.  Ref: 9278429 J Biol Chem, 1997
- In this study  **MutL**  is shown to enhance the unwinding activity of DNA helicase II  [ **UVRD** ]  more than 10 fold on a conventional helicase substrate in which a 35 residue oligonucleotide is annealed to a M13 circular single stranded phage DNA under conditions where the two proteins are present at approximately molar stoichiometry with respect to the substrate.  Ref: 9535910 J Biol Chem, 1998
- We also describe the alterations in the mutS,  **mutL**  and  **uvrD**  genes responsible for the mutator phenotype of hypermutable P. aeruginosa strains isolated from CF patients.  Ref: 11952911 Mol Microbiol, 2002
- Homologs of the Escherichia coli  **mutL** , S and  **uvrD**  and Streptococcus pneumoniae hexA, B genes involved in mismatch repair are known in several distantly related organisms.  Ref: 1459447 Genetics, 1992
- We have used artificially constructed heteroallelic heteroduplex molecules of bacteriophage lambda DNA to transfect Escherichia coli, and E. coli mutants deficient in various functions involved in the adenine methylation directed mismatch repair system,  **MutL** , MutS, MutH, and  **UvrD**  MutU.  Ref: 2961649 Genetics, 1987
- Repair of both heteroduplex orientations requires MutH,  **MutL** , MutS, DNA helicase II  [ **UVRD** ] , SSB, and DNA polymerase III holoenzyme, but the two substrates differ with respect to exonuclease requirements for correction.  Ref: 8389365 J Biol Chem, 1993
- The results shown here indicate that the putative P. aeruginosa mutS,  **mutL**  and  **uvrD**  genes are mutator genes.  Ref: 11952911 Mol Microbiol, 2002
- Seven out of the 11 mutator strains were found to be defective in the MMR system four mutS, two  **mutL**  and one  **uvrD** .  Ref: 11952911 Mol Microbiol, 2002
- Unwinding of both substrates in the presence of MutS,  **MutL** , DNA helicase II  [ **UVRD** ] , and single stranded DNA binding protein was mismatch dependent and initiated at the single strand break.  Ref: 9535911 J Biol Chem, 1998
- MutS and  **MutL**  dependent activation of DNA helicase II  [ **UVRD** ]  has also been demonstrated with a model substrate in which a 138 residue oligonucleotide was hybridized to a 138 nucleotide gap in an otherwise duplex 00 base pair circular DNA.  Ref: 9535910 J Biol Chem, 1998
- Displacement of the oligonucleotide requires MutS,  **MutL** , DNA helicase II  [ **UVRD** ] , and ATP and is dependent on the presence of a mismatch within the hybrid region.  Ref: 9535910 J Biol Chem, 1998
- In Test A, repair proficient strain and its uvrA counterpart are used for detection of spontaneous and UV induced mutations, while in Test B mismatch repair deficient strains mutH, mutS,  **mutL**  and  **uvrD**  are used for amplified detection of spontaneous mutations caused by replication errors.  Ref: 9675243 Mutat Res, 1998
- The mismatch repair system mutS,  **mutL**  and  **uvrD**  genes in Pseudomonas aeruginosa molecular characterization of naturally occurring mutants.  Ref: 11952911 Mol Microbiol, 2002
- The growth defect and filamentation phenotypes associated with 2 aminopurine exposure are effectively suppressed by null mutations in mutH,  **mutL** , mutS, or  **uvrD**  mutU, which encode activities that act upstream of the four exonucleases in the mechanism for the methyl directed reaction that has been proposed based on in vitro studies.  Ref: 11381137 Proc Natl Acad Sci U S A, 2001
- To investigate the effects on PV rates of mutations in genes for mismatch repair MMR, insertion deletion mutations of mutS,  **mutL** , mutH, dam, polI,  **uvrD** , mfd and recA were constructed in Hi strain Rd.  Ref: 11889052 EMBO J, 2002
- The Escherichia coli methyl directed reaction has been reconstituted in a purified system consisting of MutH,  **MutL** , and MutS proteins, DNA helicase II  [ **UVRD** ] , single strand DNA binding protein, DNA polymerase III holoenzyme, exonuclease I, DNA ligase, along with ATP adenosine triphosphate, and the four deoxynucleoside triphosphates.  Ref: 2665076 Science, 1989
